# Short-Chain Fatty Acids Promote Intracellular Bactericidal Activity in Head Kidney Macrophages From Turbot (*Scophthalmus maximus* L.) *via* Hypoxia Inducible Factor-1α

**DOI:** 10.3389/fimmu.2020.615536

**Published:** 2020-12-23

**Authors:** Jinjin Zhang, Hui Zhang, Miao Liu, Yawen Lan, Huiyuan Sun, Kangsen Mai, Min Wan

**Affiliations:** ^1^ Key Laboratory of Aquaculture Nutrition and Feed, Ministry of Agriculture & Key Laboratory of Mariculture, Ministry of Education, College of Fisheries, Ocean University of China, Qingdao, China; ^2^ Pilot National Laboratory of Marine Science and Technology, Qingdao, China

**Keywords:** butyrate, propionate, acetate, lysozyme, histone deacetylase, reactive oxygen species

## Abstract

Short-chain fatty acids (SCFAs) are mainly produced by microbiota through the fermentation of carbohydrates in the intestine. Acetate, propionate, and butyrate are the most abundant SCFA metabolites and have been shown to be important in the maintenance of host health. In this study, head kidney macrophages (HKMs) were isolated and cultured from turbots. We found that the antibacterial activity of HKMs was increased after these cells were incubated with sodium butyrate, sodium propionate or sodium acetate. Interestingly, our results showed that all three SCFAs enhanced the expression of hypoxia inducible factor-1 α (HIF-1α) in HKMs, and further study confirmed that butyrate augmented the oxygen consumption of these cells. Moreover, HIF-1*α* inhibition diminished the butyrate-promoted intracellular bacterial killing activity of macrophages, and SCFAs also raised the gene expression and activity of lysozymes in HKMs *via* HIF-1*α* signaling. In addition, our results suggested that butyrate induced HIF-1*α* expression and the bactericidal activity of HKMs through histone deacetylase inhibition, while G protein-coupled receptors did not contribute to this effect. Finally, we demonstrated that butyrate induced a similar response in the murine macrophage cell line RAW264.7. In conclusion, our results demonstrated that SCFAs promoted HIF-1*α* expression *via* histone deacetylase inhibition, leading to the enhanced production of antibacterial effectors and increased bacterial killing of macrophages.

## Introduction

Butyrate, propionate, and acetate, which are collectively called short-chain fatty acids (SCFAs), are products of the microbial fermentation of dietary fiber in the gut. It is well-known that SCFAs are important energy and signaling molecules, displaying beneficial effects on various physiological processes (1). In aquaculture, SCFAs have been used as growth promoters (2). Recently, the effects of SCFAs and their salts have been highlighted as immune stimulators on the health of aquatic organisms (2, 3). For example, Tian et al. demonstrated that the growth and intestinal immune functions of grass carp (*Ctenopharyngodon idella*) were improved when fish were fed a butyrate-supplemented diet, and fish receiving butyrate in their diet were protected from *Aeromonas hydrophila* infection (4). Moreover, a recent study from our research group reported that butyrate supplementation in turbot diet significantly alleviated high-soybean meal-induced enteritis (5).

In mammals, it has been demonstrated that SCFAs exert physiological functions either through the inhibition of histone deacetylases (HDACs) or the activation of G-protein-coupled receptors (GPCRs) (6). Histone deacetylation is mediated by HDACs, while HDAC inhibition induces histone hyperacetylation and reactivates suppressed genes (7). SCFAs are well-known HDAC inhibitors that have been shown to regulate the expression of numerous genes (8). Additionally, four GPCRs, GPR41, GPR43, GPR109A and Olfactory receptor 78 have been reported to mediate the different functions of SCFAs in higher animals. However, these receptors are still unknown in aquatic animals (3).

The head kidney of teleosts has been considered as a haemopoietic organ similar to the bone marrow of higher vertebrates, and it has been found that erythrocytes and leukocytes, such as macrophages, granulocytes and B lymphocytes, develop and differentiate in the teleost head kidney (9, 10). It is well-known that macrophages play a significant role in non-specific defense mechanisms in all vertebrates against invading pathogens. They have the capacity to kill pathogens through phagocytosis, production of reactive oxygen and nitrogen intermediates, and some antibacterial components, including lysozyme and antibacterial peptides (11). Therefore, head kidney macrophages (HKMs) of turbots have been isolated and utilized in our study to investigate the mechanism how SCFAs regulate the bactericidal activity of fish macrophages.

It is well-known that, hypoxia-inducible factors are the major signaling molecules that coordinate transcriptional responses to low-O_2_ environments (12). Previous studies have shown that HIF-1*α* is essential for myeloid cell function and inflammatory responses (13), and Kelly et al. also emphasized the importance of HIF-1α stabilization by SCFAs in intestine homeostasis (14). Moreover, commensal bacteria induce HIF-1*α* expression, resulting in the activation of innate immune effectors to prevent *Candida albican*s colonization (15). In this study, we have affirmed that butyrate causes increased oxygen consumption and HIF-1*α* expression in HKMs. Furthermore, we provided evidence that SCFAs promote HIF-1*α* expression *via* HDAC inhibition in HKMs, leading to the elevated production of antimicrobial effectors and the suppression of bacterial survival in macrophages. Our study, for the first time, depicts a molecular mechanism describing how SCFAs promote bacterial clearance by macrophages in fish.

## Materials and Methods

### Fish

Turbots (*Scophthalmus maximus* L.) of about 400–600 g in size were obtained from a commercial fish farm in Shandong Province, China. The fish were acclimatized in a seawater circulation system located in the Fisheries College of Ocean University of China for two weeks before the experiments. The fish were maintained in tanks (200 L) supplied with filtered, well-oxygenated and thermo-regulated seawater (salinity 35‰, temperature 17 ± 1°C), and were fed twice daily with commercial diets. No illness signs in these fish were observed during experiments. Husbandry and handling of the fish in the present study were performed strictly according to the Management Rule of Laboratory Animals (Chinese order no. 676 of the State Council, revised 1 March, 2017).

### Reagents

L-15 leibovitz cell culture medium and Dulbecco’s modified eagle medium (DMEM) with high glucose were from HyClone (Logan, Utah, USA); FBS was obtained from Invitrogen (Carlsbad, CA, USA); antibiotics (penicillin, streptomycin, and amphotericin B), PBS, Percoll and trypan blue were from Solarbio (Beijing, China); sodium acetate, sodium propionate, sodium butyrate, and trichostatin A were purchased from Sigma (St. Louis, MO, USA); antibody against HIF-1*α*, dimethyl-bisphenol A and chrysin were from Santa Cruz (Santa Cruz, CA, USA); BBoxiProbe™ R01 kit was from BestBio (Shanghai, China); RNAeasy™ animal RNA isolation kit, reactive oxygen species assay kit and DAF-FM DA were purchased from Beyotime (Shanghai, China); HiScript^®^ III RT SuperMix for qPCR was from Vazyme (Nanjing, China); SYBR green qPCR kit was from Accurate Biology (Hunan, China); Pertussis toxin was obtained from APE×BIO (Houston, Texas, USA); Lysozyme assay kit was from Jiancheng (Nanjing, China).

### HKM Isolation

Macrophages were isolated according to the method described by Chung and Secombes (16). Briefly, the head kidney of the turbot was removed and immediately washed twice with L-15 medium supplemented with penicillin (100 KU/ml), streptomycin (10 mg/ml), and amphotericin B (25 μg/ml), before being cut into small pieces. Next, the small pieces were passed through a 100 μm nylon mesh, and the obtained cell suspension was washed twice in L-15 cell culture medium with antibiotics (penicillin, streptomycin, and amphotericin B) and 2% FBS, and centrifuged at 200 g for 5 min between washes. After that, the cell suspension was separated on a 34/51% Percoll density gradient by centrifugation at 400 g. After 30 min, the cells at the interface were collected and washed twice by centrifugation at 200 g for 5 min. The cell pellets were re-suspended in L-15 medium supplemented with antibiotics, and the viability of the cells was measured by trypan blue exclusion, indicating that over 90% of the total number of cells were viable (data not shown). The cells were dispensed and cultured on different cell culture plates at 24°C. After 2 h, the non-adherent cells were washed off, and the adherent macrophages were kept in complete medium for further use.

The quality and purity of the adherent monolayers were further examined by Giemsa staining (**Supplementary Figure 1A**). When these adherent cells were analyzed by FACS (FC500, Beckman, USA), only one cell population was detected (**Supplementary Figure 1B**). Further analysis confirmed that the gene expression of macrophage colony-stimulating factor receptor (M-CSFR), a specific marker of macrophages (17), was much higher in adherent cells than in non-adherent cells (**Supplementary Figure 1C**).

### Bacterial Killing Assay

HKMs were incubated with SCFAs or additional reagents for 24 h, followed by washing three times with PBS. Afterwards, cells were incubated with equal numbers of *Edwardsiella tarda* (*E. tarda*) at 24°C under shaking. After 2 h, the cells were lysed in ice-cold water and then vortexed for 30 s. Thereafter, the cell lysates were serially diluted and plated on agar plates overnight at 28°C. The following day, viable bacteria were counted, and the bacterial survival rate was calculated.

### RNA Isolation and cDNA Transcription

RNA was extracted with the RNAeasy™ Animal RNA isolation kit according to the manufacturers’ instructions. The quantity of total RNA was determined by using a NanoDrop spectrophotometer (NanoDrop Technologies), and the quality of the extracted RNA was determined by agarose gel electrophoresis. cDNA was synthesized from total RNA using HiScript III reverse transcriptase.

### Real-Time Quantitative PCR

The sequences of all primers used in this study are listed in **Table 1**. Real-time qPCR was performed using a thermo-cycler CFX96 instrument (BioRad). The expression of target genes was normalized to *β*-actin.

**Table 1:** Primer sequences used for gene expression analysis.

Target genes	Forward primers (5′–3′)	Reverse primers (5′–3′)
Turbot *M-SCFR*	CTCCAATCAGAGGGCACCCAT	TCGGAACTGTCTCCCGTCCTT
Turbot *β-actin*	GCGTGACATCAAGGAGAAGC	TGGAAGGTGGACAGGGAAGC
Turbot *HIF-1α*	CCACCACCACTGACGATTCA	GCTGGGGTAGCTGTTGACAT
Turbot *g-type lysozyme* Murine *β-actin* Murine *HIF-1α*	GAGACTGGAACCCACACAGGAACG CATTGTTACCAACTGGGACGACA GTGAACCCATTCCTCATCCGTCA	CTGCTCTCCGCTCC AATCAGGAA GTCATCTTTTCACGGTTGGCCTT TGGCAAGCATCCTGTACTGTCC
Murine *lysozyme*	CTGGGACTCCTCCTGCTTTCT	GGGATCTCTCACCACCCTCTT
Murine *CAMP*	ACGAGGATCCAGATACTCCCAAGT	TTCCTTGAAGGCACATTGCTCAGG

### Real-Time O_2_ Consumption

The real-time O_2_ consumption in these cells was measured using a BBoxiProbe™ R01 kit according to the manufacturers’ instructions. Briefly, HKMs were cultured in a 96-well plate with a transparent bottom and black sides for 24 h. Next, 150 μl of complete medium containing sodium butyrate (NaB, 10 mM), and 10 μl of oxygen fluorescent probe was added. Meanwhile, 100 μl of blocking buffer was immediately added to each well to prevent external oxygen generation. After that, the plate was put in a microplate reader (FLUOstar Omega, BMG, Germany), and fluorescence was detected over 2 h at 2-min intervals. As the fluorescence of this oxygen probe can be quenched by O_2_, the value of the fluorescence signal was inversely proportional to the amount of O_2_ in each sample. The rate of oxygen consumption was calculated based on the changes of fluorescence signal over 2 h as follows: oxygen consumption rate (%) = (final fluorescence in NaB-treated cells − initial fluorescence in NaB-treated cells)/(final fluorescence in control cells − initial fluorescence in control cells) × 100%.

### Reactive Oxygen Species Assay

Reactive oxygen species (ROS) content was measured by using a ROS assay kit according to the manufacturer’s instructions. Briefly, HKMs were cultured in a 96-well plate with a transparent bottom and black sides for 24 h. Thereafter, the cells were treated with NaB (10 mM) or control buffer for another 24 h. After the cells were washed with PBS three times, they were co-incubated with *E. tarda* (1:1) or control buffer for 2 h. After washing with PBS, the fluorescent probe DCFH-DA was added and incubated with cells for 30 min in the dark. After washing off redundant fluorescent probe, the fluorescence values in the cells were determined using a microplate reader (FLUOstar Omega, BMG, Germany). The wells without a fluorescent probe were set as a baseline control, while the wells containing cells treated with only control buffer were set as a negative control. ROS (%) = (fluorescence in the experimental well − fluorescence in the baseline control well)/(fluorescence in the negative control well − fluorescence in the baseline control well) × 100%.

### Nitric Oxide Assay

Nitric oxide (NO) production in HKMs was analyzed using a DAF-FM DA kit according to the manufacturer’s instructions. HKMs were cultured in a 96-well plate and incubated with NaB (10 Mm) for 24 h. After washing with PBS three times, the cells were co-incubated with *E. tarda* (1:1) or control buffer for 2 h. The cells were washed again and then incubated with the fluorescent probe DAF-FM DA for 30 min in the dark. After the redundant fluorescent probe was removed, the fluorescence value of each well was determined using a microplate reader (FLUOstar Omega, BMG, Germany). The wells without the addition of a fluorescent probe were set as a baseline control, while wells containing cells treated with only control buffer were set as a negative control. NO (%) = (fluorescence in the experimental well − fluorescence in the baseline control well)/(fluorescence in the negative control well − fluorescence in the baseline control well) × 100%.

### Measurement of Lysozyme Activity

Lysozyme activity was measured with a lysozyme assay kit according to the manufacturers’ instructions. Briefly, HKMs or RAW264.7 cells were cultured in a 96-well plate overnight. After that, HKMs or RAW264.7 cells were treated with different reagents for 24 h at 24°C or 37°C, followed by three PBS washes. Next, 100 μl of cold micrococcus (100 μg/ml) was added in each well, and incubated for another 5 min. Finally, the absorbance was measured at 530 nm twice at a 2-min interval. The lysozyme activity was calculated as follows:

$$\text{Lysozyme activity }(\text{U/ml})=\text{standard activity}\left(200\text{U/ml}\right)\times \text{dilution ratio}\times (\text{final transparence in the experimental well-initial transparence in the experimental well})/\left(\text{final transparence in the standard well-initial transparence in the standard well}\right);\text{Transparence}=1/10^{\text{absorbance}}$$

### Statistical Analysis

Results are presented as the mean ± SEM. Differences between the means were evaluated using one-way ANOVA or Tukey’s t-test. *P <* 0.05 was considered statistically significant.

## Results

### SCFAs Enhanced the Bactericidal Activity of Turbot HKMs

**Figure 1:**
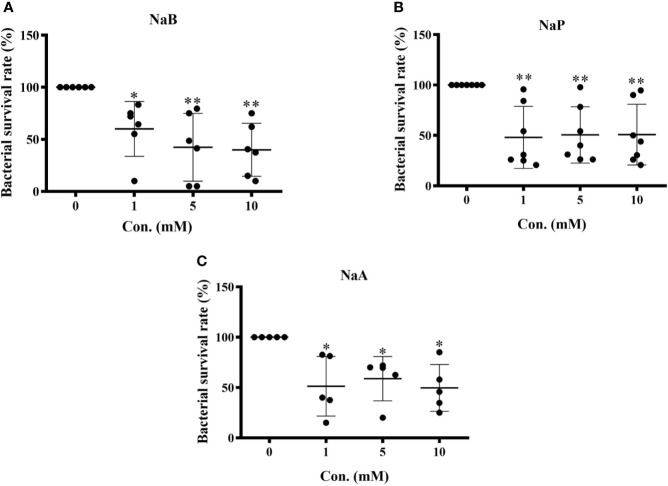
Short chain fatty acids (SCFAs) restrained bacterial growth in turbot head kidney macrophages (HKMs). HKMs were incubated with **(A)** sodium butyrate (NaB, n = 6), **(B)** sodium propionate (NaP, n = 7) and **(C)** sodium acetate (NaA, n = 5) in different concentrations (0, 1, 5, 10 mM) for 24 h. Thereafter, macrophages were subjected to *Edwarsiella tarda* (1:1) for 2 h, and the survival rate of ingested bacteria in SCFAs-treated macrophages was measured as described in Methods. The results were from at least three independent experiments. Error bars are presented as mean ± SD; *p < 0.05, **p < 0.01.

To assess the influence of SCFAs on the bacterial killing ability of turbot HKMs, isolated macrophages were incubated with control buffer, sodium butyrate (NaB), sodium propionate (NaP) or sodium acetate (NaA) at different concentrations (1, 5, 10 mM) for 24 h. Thereafter, macrophages were subjected to *E. tarda* for 2 h, and the number of viable bacteria in the macrophages were counted. As the results showed, the survival rates of *E. tarda* in HKMs pretreated with NaB (**Figure 1A**), NaP (**Figure 1B**) or NaA (**Figure 1C**) were significantly lower than those in macrophages treated with control buffer. These findings suggest that all three SCFAs promote the bactericidal activity of turbot HKMs.

### SCFAs Augmented Oxygen Consumption and HIF-1α Expression in Turbot HKMs

**Figure 2:**
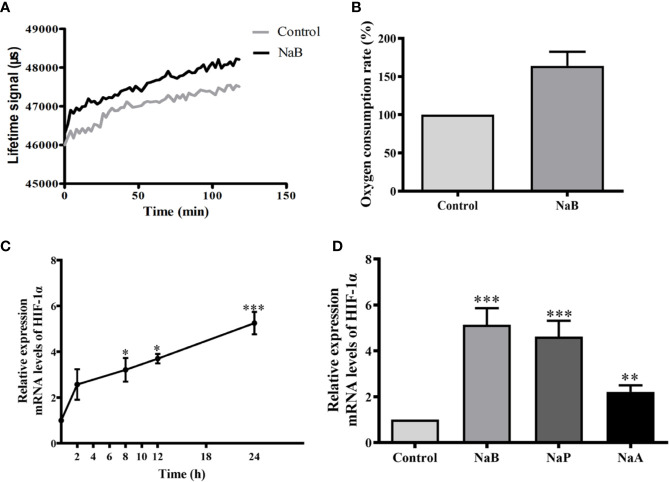
SCFAs elevated oxygen consumption and HIF-1*α* gene expression in HKMs. **(A, B)** When HKMs were treated with NaB (10 mM), fluorescence of the oxygen probe in the cells was monitored within 2 h **(A),** and the rate of oxygen consumption at 2 h in HKMs was analyzed (**B**, n = 3). **(C)** Isolated macrophages were incubated with NaB (10 mM) for different time courses (0, 2, 8, 12, and 24 h), and HIF-1*α* gene expression in macrophages was detected (n = 3). **(D)** After HKMs were stimulated with 10 mM of NaB (n = 11), NaP (n = 6) or NaA (n = 6) for 24 h, the gene expression of HIF-1*α* in macrophages was measured. The results were representative of at least three independent experiments, and data were normalized by comparing to the control group. Error bars represent mean ± SEM. *p < 0.05, **p < 0.01, ***p < 0.001.

To test whether SCFAs treatment affected the oxygen consumption by these cells, the oxygen content in turbot HKMs was monitored. Our results showed that when HKMs were subjected to NaB, more fluorescent O_2_ sensor was detected in these cells than in control buffer-treated cells, since O_2_ could quench the fluorescence of O_2_ sensor (**Figure 2A**). Our results also suggest that NaB induces a rapid oxygen consumption in macrophages. Around 50% more oxygen consumption was detected after HKMs were treated with NaB for 2 h (**Figure 2B**).

Since HIF-1*α* expression and stability is strictly regulated by oxygen stress, the gene expression of HIF-1*α* was analyzed in SCFA-treated HKMs. When macrophages were exposed to NaB for different times up to 24 h, the results showed that the gene expression of HIF-1*α* increased steadily from 0 to 24 h (**Figure 2C**). Moreover, HIF-1*α* gene expression in HKMs treated with NaB, NaP or NaA for 24 h was elevated significantly compared to the control group (**Figure 2D**).

### SCFAs Regulated the Production of Antibacterial Effectors in Turbot HKMs

**Figure 3:**
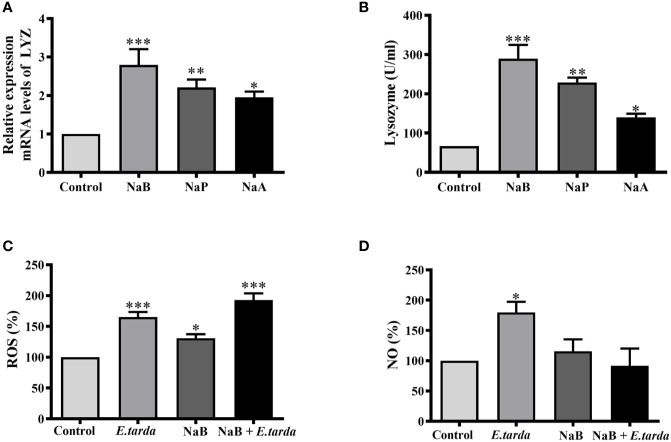
SCFAs promoted the gene expression and enzyme activity of lysozyme. **(A, B**) HKMs were stimulated with SCFAs (10 mM) for 24 h to analyze the gene expression of lysozyme (**A**, n = 7) and enzyme activity (**B**, n = 4). **(C, D)** HKMs were treated with NaB (10 mM) for 24 h. Thereafter, *Edwarsiella tarda* was added (cells: bacteria *=* 1:1) and co-incubated for 2 h, and the contents of ROS (**C,** n = 8) or NO (**D,** n = 8) in the cells were measured. The results were calculated from at least three independent experiments; error bars were presented as mean ± SEM. *p < 0.05, **p < 0.01, ***p < 0.001.

To identify which effectors could contribute to SCFA-boosted intracellular bacterial killing of HKMs, the gene expression of lysozyme and enzyme activity was analyzed. As our results showed, all three SCFAs significantly raised the gene expression of lysozyme (**Figure 3A**) as well as lysozyme enzyme activity (**Figure 3B**). In addition, the production of ROS and NO in NaB-treated HKMs was also measured. The results showed that NaB incubation alone promoted ROS production, while no effect on NO production was observed in HKMs (**Figures 3C, D**). As expected, *E. tarda* infection induced the production of ROS and NO in HKMs, and NaB pretreatment further elevated *E. tarda*-induced ROS content in HKMs (**Figure 3C**). However, NaB repressed the NO production caused by *E. tarda* infection (**Figure 3D**).

### HIF-1α Mediated Butyrate-Induced Lysozyme Expression and Bactericidal Activity in Macrophages

**Figure 4:**
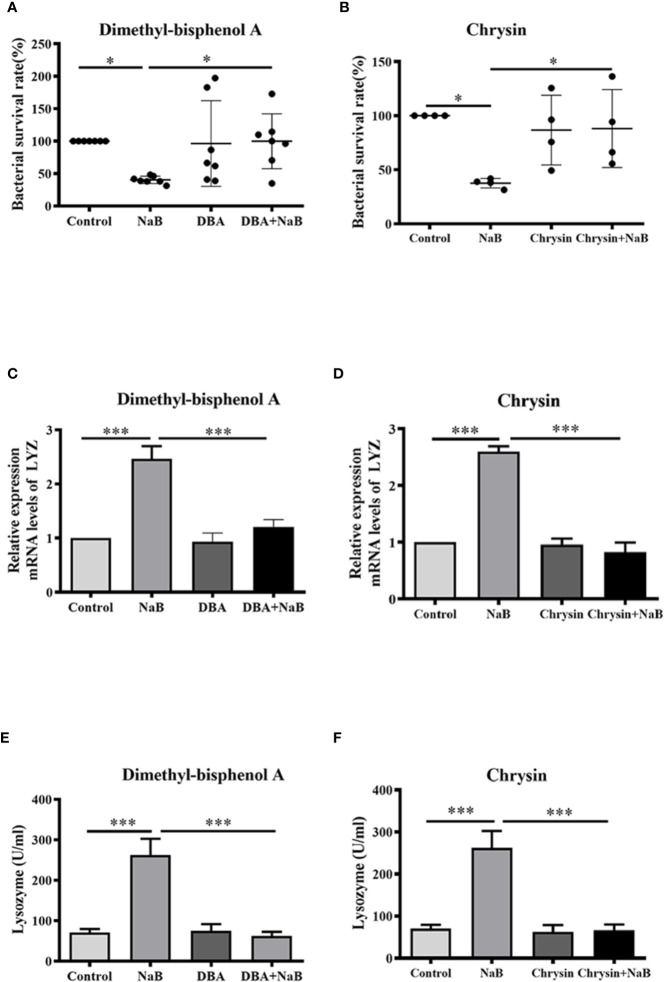
Butyrate increased bactericidal activity of macrophages through activation of HIF-1*α*. **(A, B)** HKMs were exposed to NaB (1 mM) as well as specific HIF-1α inhibitor, dimethyl-bisphenol A (DBA, 25 μM) (**A**, n = 7) or chrysin (25 μM) (**B**, n = 4) for 24 h, followed by the assessment of bacterial killing activity of treated macrophages. Error bars are presented as mean ± SD. **(C, D)** HKMs were treated with NaB (1 mM) plus DBA (**C,** n = 6) or chrysin (**D**, n = 3) for 24 h, and the gene expression of lysozyme was detected. **(E, F)** HKMs were incubated with NaB (1 mM) plus DBA (**E,** n = 7) or chrysin (**F**, n = 7) for 24 h, and lysozyme activity was assessed. Data were calculated from at least three independent experiments. * *p* < 0.05, ***p < 0.001.

Next, HKMs were pre-incubated with two specific HIF-1*α* inhibitors, dimethyl-bisphenol A (DBA) or chrysin, to confirm whether HIF-1*α* was associated with SCFA-induced antibacterial activity. As our results showed, butyrate almost ablated the ability to lower bacterial survival in HKMs, when HIF-1*α* activity was inhibited by DBA (**Figure 4A**) or chrysin (**Figure 4B**). Similarly, the butyrate-promoted gene expression of lysozyme (**Figures 4C, D**) and lysozyme enzyme activity (**Figures 4E, F**) was diminished in HKMs, when macrophages were pre-incubated with HIF-1*α* inhibitors. These results indicate that HIF-1*α* signaling plays a vital role in butyrate-induced intracellular bacterial killing of macrophages.

### SCFAs Activated HIF-1α Signaling and Antibacterial Mechanisms in HKMs Through HDAC Inhibition

**Figure 5:**
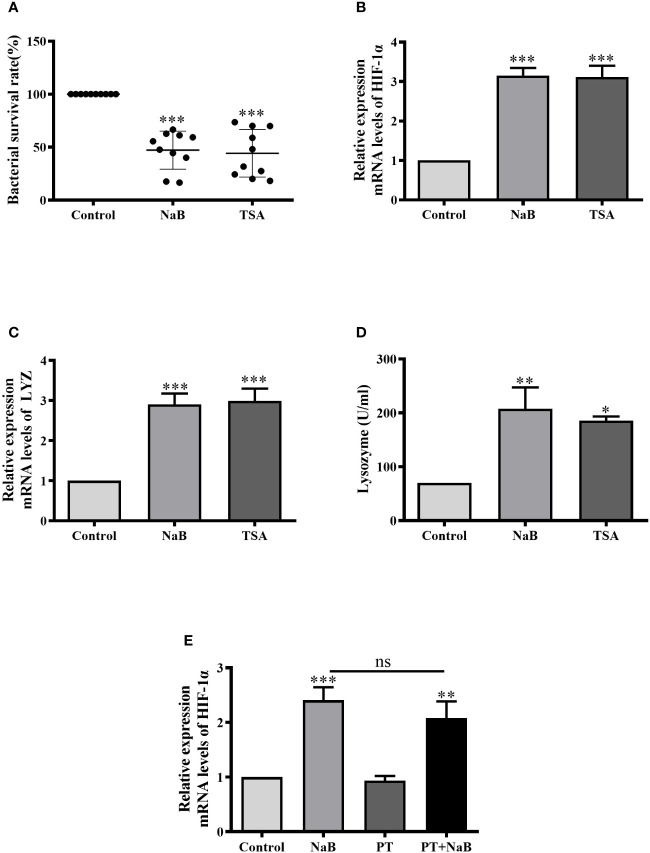
SCFAs augmented HIF-1*α* expression, lysozyme activity and bacterial killing of HKMs *via* HDAC Inhibition. **(A–D)** HKMs were treated with NaB (10 mM) or TSA (1 μM). After 24 h, the bacterial survival rate in HKMs (**A**, n = 10), the gene expression of HIF-1*α* (**B**, n = 8) or lysozyme (**C**, n = 7), and lysozyme activity (**D**, n = 4) were analyzed. **(E)** HKMs were treated with control buffer, NaB (1 mM), pertussis toxin (PT, 1 μg/ml) or NaB plus PT for 24 h, and the gene expression of HIF-1*α* in HKMs was assessed (n = 14). The results were calculated from at least three independent experiments. *p < 0.05, **p < 0.01, ***p < 0.001, ns: non significance.

In the following experiments, we attempted to clarify if butyrate activates HIF-1*α* either through HDAC inhibition or *via* GPCRs. First, we tested the effects of trichostatin A (TSA), a well-known HDAC inhibitor. Consistently, the bacterial survival rate in HKMs was significantly lower in TSA or NaB-treated macrophages than in control cells (**Figure 5A**). In addition, TSA also significantly enhanced the gene expression of HIF-1*α* in HKMs, as NaB did (**Figure 5B**). Moreover, lysozyme gene expression, as well as lysozyme enzyme activity, increased in TSA-treated HKMs (**Figures 5C, D**).

In contrast, the effects of pertussis toxin (PT), a specific inhibitor of GPCR signaling, were assessed. When HKMs were co-treated with NaB and PT, PT displayed no effects on NaB-induced HIF-1*α* expression compared to the cells treated with butyrate alone (**Figure 5E**), confirming that GPCRs were not involved in this response.

### Butyrate Enhanced Antibacterial Activity in Murine RAW264.7 Cells

**Figure 6:**
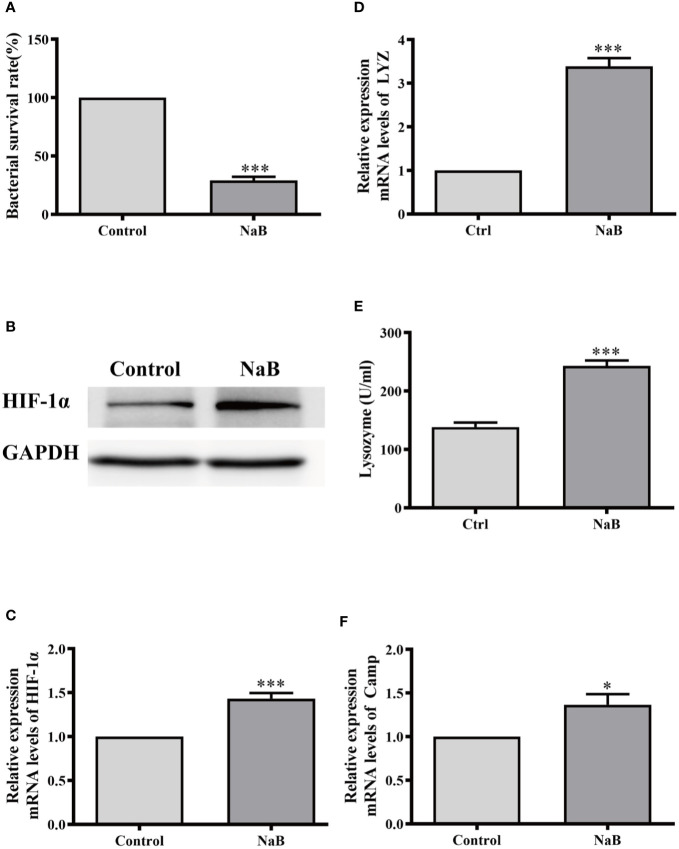
Sodium butyrate elevated antibacterial activity of RAW264.7 cells. RAW264.7 cells were cultured and stimulated with control buffer or NaB (1 mM) for 24 h. Afterwards, **(A)** the bacterial survival rate in RAW264.7 cells was analyzed (n = 8); **(B, C)** the protein level of HIF-1α in HKMs was assessed **(B)** and HIF-1α gene expression was also detected (**C**, n = 7); **(D, E)** The gene expression of lysozyme (**D**, n = 10) and lysozyme activity (**E**, n = 6) was measured. **(F)** The *CAMP* gene expression was analyzed (n = 8). The results were calculated from at least three independent experiments. Error bars represent mean ± SEM. *p < 0.05, ***p < 0.001.

We further examined whether butyrate induced the same effects on macrophages from other species. Therefore, the murine macrophage cell line RAW264.7 was cultured and incubated with NaB for 24 h. In agreement with our results from HKMs, the bacterial survival rate in butyrate-treated RAW264.7 cells also significantly declined compared to control cells (**Figure 6A**). Meanwhile, butyrate elevated the gene and protein expression of HIF-1*α* (**Figures 6B, C**), and promoted lysozyme production in RAW264.7 cells (**Figures 6D, E**). As shown in **Figure 6F**, butyrate also elevated the gene expression of murine cathelicidin. Thus, the butyrate-activated antibacterial signaling pathway in macrophages seems to be conserved across evolution from teleosts to mammals.

## Discussion

In this study, we isolated and cultured HKMs from turbots, and demonstrated that SCFAs enhanced HIF-1*α* expression and the production of antimicrobial components *via* HDAC inhibition, leading to an enhanced bactericidal activity in turbot HKMs. SCFAs have been shown to promote immune responses and infectious disease resistance in fish (18, 19). Our study provides the evidence to explain on a cellular level how SCFAs contributed to the bacterial clearance by immune cells in fish.

Host microbial cross-talk has attracted a great deal of interest in recent years, and accumulating evidence has demonstrated that gut microbiota is essential to maintain intestinal homeostasis and the regulation of host health (20). SCFAs are bacterial fermentation products from dietary fibers and include mainly acetate (C2), propionate (C3) and butyrate (C4) in higher animals, as well as in aquatic animals (3). It is well-known that SCFAs are major mediators of host−microbe interaction in the intestine (1), and SCFAs, especially butyrate, promote intestinal epithelial barrier function and regulate the host mucosal immune system (1, 6).

In fish, the concentrations of SCFAs increase towards the distal intestine (3), and it has been reported that the total SCFAs in the hindguts of herbivorous freshwater grass carp (*Ctenopharyngodon idellus*) is approximately 5.04 mM (21), while SCFAs in the hindgut of two herbivorous marine fish, *K. sydneyanus* and *O. pullus*, were found to be higher than 37 mM (22). In addition to the gastrointestinal tract, the presence of SCFAs in the oral cavity and female genital tract of humans has been detected in the millimolar range (23, 24). Interestingly, it has also been reported that gut-microbiota-derived SCFAs can exert their influence in peripheral tissues. As an example, it was shown that gut microbiota-derived SCFAs promoted the expression of the antimicrobial peptide CRAMP in pancreatic *β*-cells to prevent the development of diabetes (25). Moreover, Trompette et al. demonstrated convincingly that mice fed with a high-fiber diet exhibited increased circulating levels of SCFAs, and these mice were protected against allergic inflammation in the lung (26). Although the plasma concentrations of SCFAs in higher animals have been reported to be in the micromolar range (26, 27), there is little information regarding the threshold levels of SCFAs needed for beneficial responses within the peripheral tissues. Nevertheless, our results show that SCFAs ranging from 1 to 10 mM promoted bacterial killing of macrophages *in vitro*. Additionally, we tested concentrations of butyrate as low as 10 μM, which also augmented the bactericidal activity of turbot HKMs (data not shown). Considering the gastrointestinal tract and other peripheral tissues harbor a large reservoir of tissue macrophages, protecting the mucosal tissues against harmful pathogens (28), it would be reasonable to speculate that microbiota-derived SCFAs could promote pathogen clearance by intestinal and other peripheral macrophages in fish and also in higher animals, since butyrate exhibited similar effects on murine macrophages (**Figure 6**).

SCFAs exert their functions *via* either GPCRs or HDAC inhibition. It is widely known that SCFAs inhibit HDAC activity in many cell types, and previous studies have shown that butyrate, and to a lesser extent, propionate acts as an HDAC inhibitor, exerting effects on the inflammatory process by downregulating the expression of pro-inflammatory genes (29–31). In addition, acetate can also act as an HDAC inhibitor (32, 33). In our study, we demonstrated that SCFAs induced HIF-1*α* expression in HKMs *via* the inhibition of HDACs. To date, the mechanism by which SCFAs inhibit HDACs is still unclear. It appears that SCFAs may directly act on HDACs *via* different transporters on the cells, or indirectly through GPCR activation (34). Our results, however, have indicated that SCFAs enter into target cells and act inside these cells, since we demonstrated that GPCRs were not involved in SCFA-induced responses (**Figure 5E**). Although diffusion is a route for SCFAs to enter cells, studies in higher animals have revealed that carrier-mediated mechanisms constitute the major route for the entry of SCFAs in their anionic form into target cells, especially in the colonic epithelium (35). So far, several transport systems responsible for the cellular uptake of SCFAs have been identified in higher animals, including H^+^-coupled and Na^+^-coupled transport system (35). However, little is known about SCFA transporters in fish. Thus, it would be very interesting to further clarify how SCFAs are transported into macrophages in teleosts.

Hypoxia-inducible factors are the major cellular mechanisms that coordinate the transcriptional responses to low-O_2_ environments (12). It was previously demonstrated that SCFAs depleted oxygen and induced the stabilization of HIF-1*α* in intestinal epithelial cells (14), which increased epithelial barrier function and reduced intestinal inflammation and bacterial translocation (36). Interestingly, it was reported that acute HDAC inhibition resulted in rapid increased oxygen consumption in the head tissue of the fruitfly (37) and in diabetic mice (38). Consistently, our study confirmed that oxygen consumption was increased significantly in butyrate-incubated macrophages, which led to enhanced HIF-1α expression in turbot HKMs (**Figure 2**).

More importantly, HIF-1*α* is a critical hub that integrates hypoxic and immunogenic signals during infection and/or inflammation (39). Previously it was reported that HIF-1*α* expression in murine macrophages and neutrophils is essential for effective bacterial killing *via* promoting the production of key immune effector molecules, such as antimicrobial components, NO and TNF-α (40). Lysozyme is considered to be a key component of the innate immune response to pathogen infections and has strong antibacterial activity. It is well documented that fish lysozyme possesses lytic activity against both Gram-positive bacteria and Gram-negative bacteria (41). The g-type lysozyme in turbot has been shown to play an important role in the defense against most bacterial infections (42), in particular, g-type lysozyme increased the disease resistance in the mucosal surfaces of fish (43). Therefore, the expression and activity of turbot g-type lysozyme in HKMs were analyzed in our study. Our results showed that the expression and activity of g-type lysozymes in butyrate-treated turbot HKMs was elevated in a HIF-1*α*-dependent manner, which potentially contributes to the SCFA-promoted bacterial killing of macrophages.

It is well-known that ROS have potent antimicrobial activity against bacteria, fungi and viruses (44). The primary sources of ROS in phagocytes come from “respiratory burst” by conversion of O_2_ to O_2_^-^ *via* oxidases and subsequent dismutation to H_2_O_2_ (45). Previous report has exhibited a dependence of ROS production on oxygen consumption in cells (46). Thus, our results indicate that butyrate increases oxygen consumption in macrophages, resulting in the enhanced ROS generation, as well as the upregulation of HIF-1*α*-dependent lysozyme production. Consequently, butyrate-promoted antimicrobial effectors, including ROS and lysozymes, contribute to eliminate intracellular bacteria in macrophages. In addition, NO is a small messenger that regulates a variety of physiological functions, including phagocytic defense mechanisms (47). In fact, NO has been recognized as one of the most versatile players in the immune system (48). Previous reports have demonstrated that butyrate inhibits bacteria-induced NO production in murine macrophages (29, 49), and our result provides the evidence that butyrate causes a similar response in HKMs (**Figure 3D**).

Antimicrobial peptides (AMPs) are another important set of factors in host defense against pathogenic microbes. In fish, cationic AMPs are mainly divided into five families, including piscidins, cathelicidins, defensins, hepcidins and high-density lipoproteins (50). Previous study has illustrated that the activation of HIF-1*α* resulted in the elevated expression of cathelicidins in order to inhibit gastrointestinal colonization of fungi (15). Recently, it was reported that the activation of intestinal HIF-1*α* boosted local AMP expression to facilitate microbial homeostasis in zebrafish (51). Nevertheless, our result showed that the gene expression of turbot hepcidin in HKMs was not increased by SCFA treatment (data not shown). Although cathelicidins and *β*-defensins, two important AMP families, have not been identified in turbot, and the result from murine macrophages showed that butyrate significantly upregulated the gene expression of cathelicidin antimicrobial peptide (**Figure 6F**), which might be beneficial to butyrate-promoted bactericidal activity of macrophages.

## Conclusion

**Figure 7:**
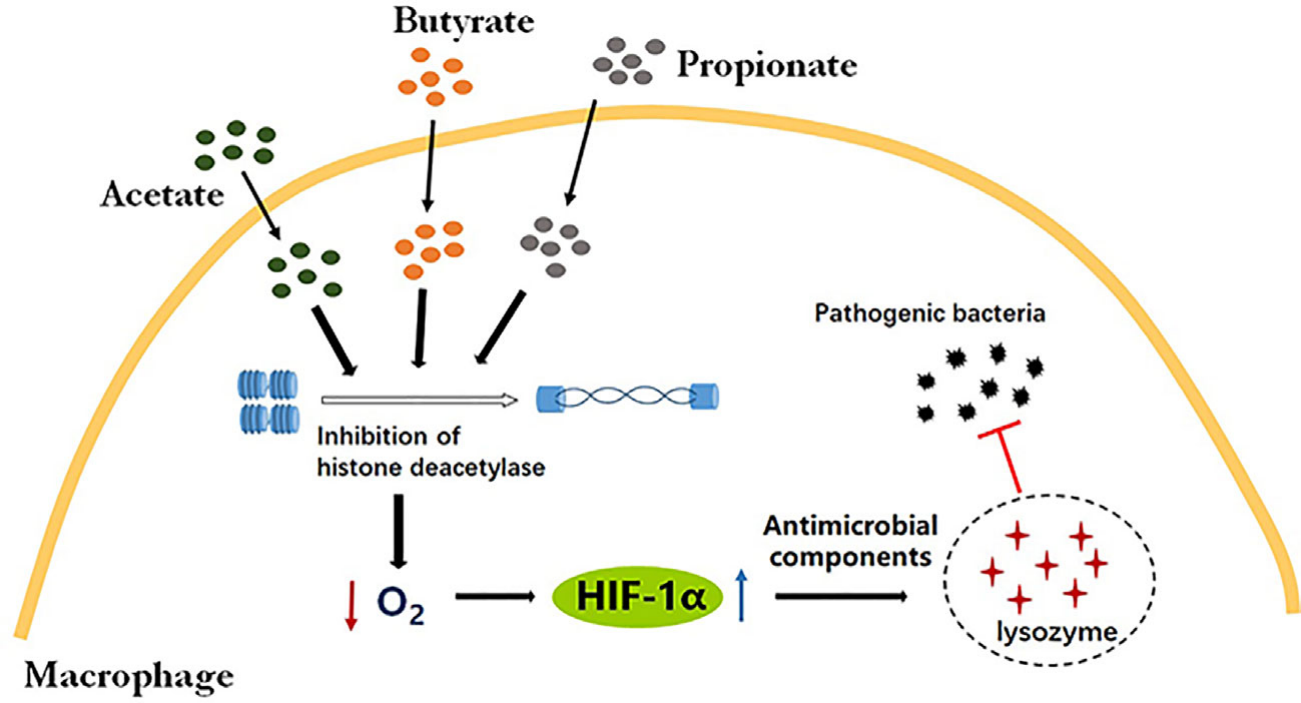
SCFAs promoted intracellular bactericidal activity of macrophages *via* HIF-1*α*. SCFAs, including butyrate, propionate and acetate, induce hypoxia and HIF-1*α* expression *via* HDAC inhibition in macrophages. The activation of HIF-1*α* signaling enhances the production of antibacterial components, such as lysozymes, leading to the efficient clearance of pathogenic bacteria in macrophages.

For the first time, our study has demonstrated that SCFAs augmented oxygen consumption and activated HIF-1*α* signaling *via* HDAC inhibition in macrophages. Moreover, SCFA-induced HIF-1*α* resulted in the elevated production of antimicrobial effectors and bacterial clearance by macrophages, revealing potential novel mechanisms whereby SCFAs contribute to bacterial clearance by macrophages (**Figure 7**).

## Data Availability Statement

The raw data supporting the conclusions of this article will be made available by the authors, without undue reservation.

## Ethics Statement

The animal study was reviewed and approved by the Institutional Animal Care and Use Committee of the Ocean University of China. The present study was conducted in strict accordance with the recommendations in the Guide for the Use of Experimental Animals of Ocean University of China. All efforts had been dedicated to minimize suffering of the animals.

## Author Contributions

JZ designed and performed experiments, analyzed data and wrote the manuscript. HZ, ML, YL, and HS performed experiments. KM supervised the project. MW supervised the project, designed experiments, analyzed data, and wrote the manuscript. All authors contributed to the article and approved the submitted version.

## Funding

This study was supported by National Key R&D Program of China (Grant No. 2018YFD0900400); the National Natural Science Foundation of China (Grant No. 31972802); Natural Science Foundation of Shandong Province (Grant No. ZR2019MC041); Youth Talent Program Supported by Laboratory for Marine Fisheries Science and Food Production Processes, Pilot National Laboratory for Marine Science Technology (Qingdao) (Grant No. 2018-MFS-T11).

## Conflict of Interest

The authors declare that the research was conducted in the absence of any commercial or financial relationships that could be construed as a potential conflict of interest.
