# Supplementary material for: Short-Chain Fatty Acids Promote Intracellular Bactericidal Activity in Head Kidney Macrophages From Turbot (Scophthalmus maximus L.) via Hypoxia Inducible Factor-1α
Source: Front Immunol. 2020 Dec 23;11:615536. doi: 10.3389/fimmu.2020.615536 (PMC7785818; doi:10.3389/fimmu.2020.615536)
Supplement: Supplementary file 1 [file DataSheet_1.pdf]

A

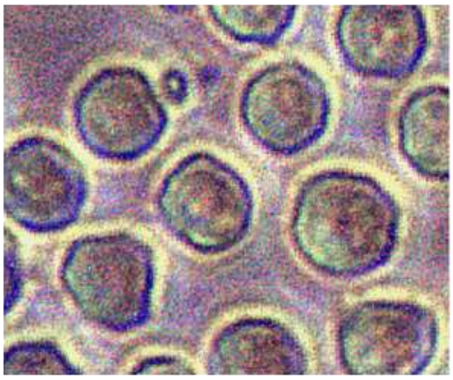

B

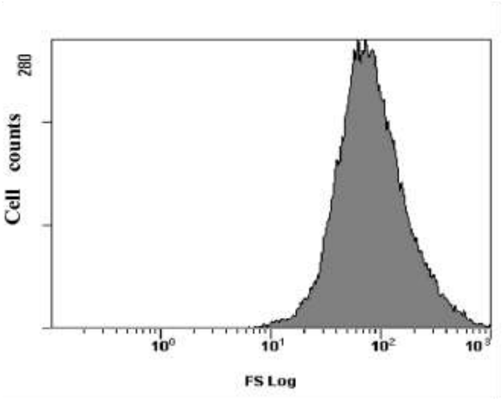

C

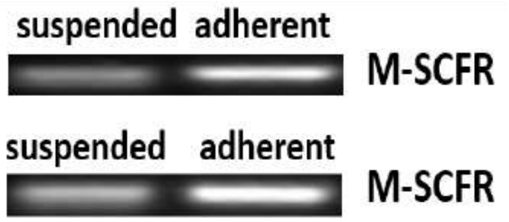

Supplemental Figure 1. The morphology and characterization of HKMs. (A-B) The adherent monolayer was examined by Giemsa staining **(A)** or analyzed by flow cytometry (**B**). (**C**) The non-adherent and adherent cells were collected respectively, and the gene expression of macrophage colony-stimulating factor receptor (M-CSFR) in two groups was detected by PCR. The figure was representative of at least three independent experiments.
